# Implementation of clinical tractography for pre-surgical planning of space occupying lesions: An investigation of common acquisition and post-processing methods compared to dissection studies

**DOI:** 10.1371/journal.pone.0231440

**Published:** 2020-04-14

**Authors:** Jonathan Ashmore, Hugh G. Pemberton, William D. Crum, Jozef Jarosz, Gareth J. Barker

**Affiliations:** 1 Institute of Psychiatry, Psychology and Neuroscience, King's College London, Institute of Psychiatry, London, England, United Kingdom; 2 Department of Neuroradiology, King’s College Hospital NHS Foundation Trust, London, England, United Kingdom; NIH, UNITED STATES

## Abstract

**Background and purpose:**

There is limited standardization of acquisition and processing methods in diffusion tractography for pre-surgical planning, leading to a range of approaches. In this study, a number of representative acquisition variants and post processing methods are considered, to assess their importance when implementing a clinical tractography program.

**Methods:**

Diffusion MRI was undertaken in ten healthy volunteers, using protocols typical of clinical and research scanning: a 32-direction diffusion acquisition with and without peripheral gating, and a non-gated 64 diffusion direction acquisition. All datasets were post-processed using diffusion tensor reconstruction with streamline tractography, and with constrained spherical deconvolution (CSD) with both streamline and probabilistic tractography, to delineate the cortico-spinal tract (CST) and optic radiation (OR). The accuracy of tractography results was assessed against a histological atlas using a novel probabilistic Dice overlap technique, together with direct comparison to tract volumes and distance of Meyer’s loop to temporal pole (ML-TP) from dissections studies. Three clinical case studies of patients with space occupying lesions were also investigated.

**Results:**

Tracts produced by CSD with probabilistic tractography provided the greatest overlap with the histological atlas (overlap scores of 44% and 52% for the CST and OR, respectively) and best matched tract volume and ML-TP distance from dissection studies. The acquisition protocols investigated had limited impact on the accuracy of the tractography. In all patients, the CSD based probabilistic tractography created tracts with greatest anatomical plausibility, although in one case anatomically plausible pathways could not be reconstructed without reducing the probabilistic threshold, leading to an increase in false positive tracts.

**Conclusions:**

Advanced post processing techniques such as CSD with probabilistic tractography are vital for pre-surgical planning. However, overall accuracy relative to dissection studies remains limited.

## Introduction

The use of diffusion imaging based tractography to inform and guide the surgical resection of space occupying lesions is becoming increasingly prevalent. This is partly due to the inclusion of tractography software into clinical neurosurgical planning systems. Early on it was recognized that tractography is limited in fully depicting the underlying anatomical structure [1], which can in part be attributed to a number of technical challenges [2, 3]. One such limitation reported in the literature is the disruption to the tracking process which can occur due to signal loss in diffusion weighted images from vascular and CSF pulsation [4, 5]. To avoid this artefact, groups have suggested that peripheral gating the DWI acquisition to the cardiac cycle should be undertaken. However, there has been limited implementation of this in the clinical setting due to increases in scan time and difficulties in patient preparation [2]. Another limitation of tractography is the inability to fully depict all white matter projections in regions of complex fibre architecture. The simplest form of tractography based on the diffusion tensor model assumes only a single fibre population within a voxel, but it has been shown more than 90% of the white matter voxels in a typical DWI acquisition will contain at least 2 fibre populations [6]. Despite this, the clinically available tractography systems seem to exclusively implement diffusion tensor based tractography. Methods to better elucidate the complex fibre architecture have been available in the research setting for some time and include approaches which model crossing fibre architecture [7–11] and probabilistic tractography [12]. Several studies have highlighted the need for clinically based systems to implement this advanced diffusion processing methodology [13, 14]. However, to successfully implement these advanced methods a thorough understanding is required of the additional acquisition requirements and processing parameters associated with the more complex modelling. One such variable is the threshold in probabilistic tractography; varying this threshold heavily influences the number of the false positive and false negative pathways included in the final tract representation [15–17]. Another issue which has been heavily discussed in the literature considers the number of diffusion encoding directions required for tractography. This optimal number has been investigated in numerous settings including by the reproducibility of quantitative metrics [18], the accuracy of anatomical depiction [19] and in the context of different diffusion modelling algorithms [20]. The literature provides varying interpretations on the optimal number of directions, making it difficult for a clinical site implementing tractography to know which protocol represents the best compromise between data quality and scan time.

With so many seemingly important technical refinements in diffusion acquisition and tractography processing it can often be daunting for an inexperienced user to know how best to initiate a program of tractography for neurosurgical planning. Incorporating all the suggested refinements can lead to long scan times and the requirement for technical expertise in acquisition setup and offline processing. To this end, the goal of the work presented here was to investigate these varying methodologies and assess their relevance in clinical tractography of the cortico-spinal tract (CST) and optic radiations (OR) for neurosurgical planning of space occupying lesions (SOLs). With this application in mind we aimed to address the following issues: [1] how tractography is influenced by the number of diffusion encoding directions collected, [2] how tractography is influenced by the use of peripheral gating, [3] whether and how the results of advanced processing methods, including constrained spherical deconvolution (CSD) and probabilistic tractography, differ from DTI based methods with streamline tractography, as implemented as standard on our neurosurgical planning system and [4] what the optimal threshold is for probabilistic based tractography in applications such as these. The purpose of this investigation was to understand where best to focus our efforts, such that an optimal tractography acquisition and processing pipeline could be implemented within our clinical setting.

All of the above methodologies have been investigated and reported previously in various settings. The novel aspect of our work is to assess the tractography results against the Jülich histological atlas [21] using a probabilistic Dice overlap technique [22] and through a direct comparison between tract volumes and anatomical location with equivalent measures reported in dissections studies [23–25]. We use this assessment to determine an optimum acquisition and processing protocol. The fuzzy Dice overlap technique has never previously been used as a metric for assessment of tractography accuracy and to the best of our knowledge dissection studies have only previously been evaluated against the OR. By comparing to a number of dissection metrics for both the CST and OR we provide the most comprehensive body of work comparing tractography methods to dissection studies for application to neurosurgical planning.

Finally we show the result of applying our suggested optimised processing protocol to a series of clinical case studies.

## Methods

### Subjects

Ten healthy volunteers (8 male, age range 23–51 years) were prospectively recruited for the study. Informed written consent was obtained with ethical approval from the London–Camberwell St Giles Research Ethics Committee. Data from three patients referred to the neurosurgical unit at Kings College Hospital, London, UK for presurgical imaging were also included retrospectively as part of a clinical audit undertaken according to King’s College Hospital standard policies and procedures (Table 1)

**Table 1:** Patient demographics.

Patient	Age	Sex	Diagnosis
1	14	M	Right peri-rolandic Ganglioglioma WHO grade I
2	57	M	Left frontal Oligodendroglioma WHO grade II
3	50	F	Left parietal arterial venous malformation (AVM)

### Acquisition protocol

MRI was performed using a 1.5T HDxt system (General Electric Healthcare, Chicago, USA) with an 8-channel head coil. Structural images were acquired with a 3D IR prepared spoiled gradient echo sequence (IRSPGR); TI = 300ms, TE = 5ms, TR = 11.6ms, Flip angle = 20, FOV = 28cm, 131 slice locations, 1.1mm isotropic voxel size, ASSET (parallel imaging) factor = 2. The DWI acquisition utilized a double refocused spin echo EPI sequence; TR = 17s, TE = 101ms, 2.5mm isotropic voxel size, 52 slices, to provide whole brain coverage. 32 diffusion encoding directions, with b-value = 1500s/cm^2^, were collected along with 4 b = 0 images (acquisition time, 10min 24s). This “base” acquisition was subsequently repeated with the following modifications applied separately: (a) 64 diffusion directions with 7 b = 0 images (acquisition time, 20min 02s); (b) peripheral gating to the cardiac cycle with effective TR = 15 R-R intervals (10min 48s). This gave a total of three raw diffusion acquisition data sets which are subsequently labelled; 32 dir (for the 32 direction acquisition), 64 dir (for the 64 direction acquisition) and 32 dir PG (for the 32 direction acquisition with peripheral gating). The decision to test 32 and 64 direction diffusion acquisition schemes was in part motivated by the work of [20] who suggests that 45 directions is the minimum to be used for processing methods which attempt to reconstruct multiple fibre populations within a voxel. Despite this, groups have successfully investigated diffusion tractography based on reconstruction algorithms that identify multiple fibre populations within a voxel for neurosurgical planning with diffusion encoding schemes of < 45 directions [26–28]. It has also been shown for DTI that at least 30 unique sampling orientations are required for a robust estimation of the tensor orientation [18]. Given these results, we opted to test a 32 direction encoding scheme (having a clinically acceptable acquisition time of 10 min) against a 64 direction encoding scheme, chosen to be consistent with reports of similarly high numbers of encoding directions [14, 29, 30]. Given the increased imaging time for the 64 direction acquisition (>20mins) a strong justification of additional utility would be required for us to implement in our clinical population.

### Diffusion processing

Raw diffusion data were initially viewed in cine mode using fslview (www.fmrib.ox.ac.uk/fsl/fslview) and it was found there was limited motion present. Retrospective motion correction could have been applied via tools incorporated into the different processing methods investigated in this study (methods described below). However, the motion correction techniques differed for each method and therefore for consistency, and since limited motion was present in our data, no correction was applied.

Each diffusion data set was post-processed using the CE marked StealthViz tractography processing software (Medtronic, Colorado, USA) which utilizes a diffusion tensor reconstruction and the FACT streamline tractography algorithm [31]. The raw diffusion data was subsequently taken offline and processed using the MRTrix software package (www.mrtrix.org). Tracts were produced using the constrained spherical deconvolution (CSD) reconstruction, with tractography subsequently implemented based on the streamline and bootstrap probabilistic algorithms [32].

**Fig 1:**
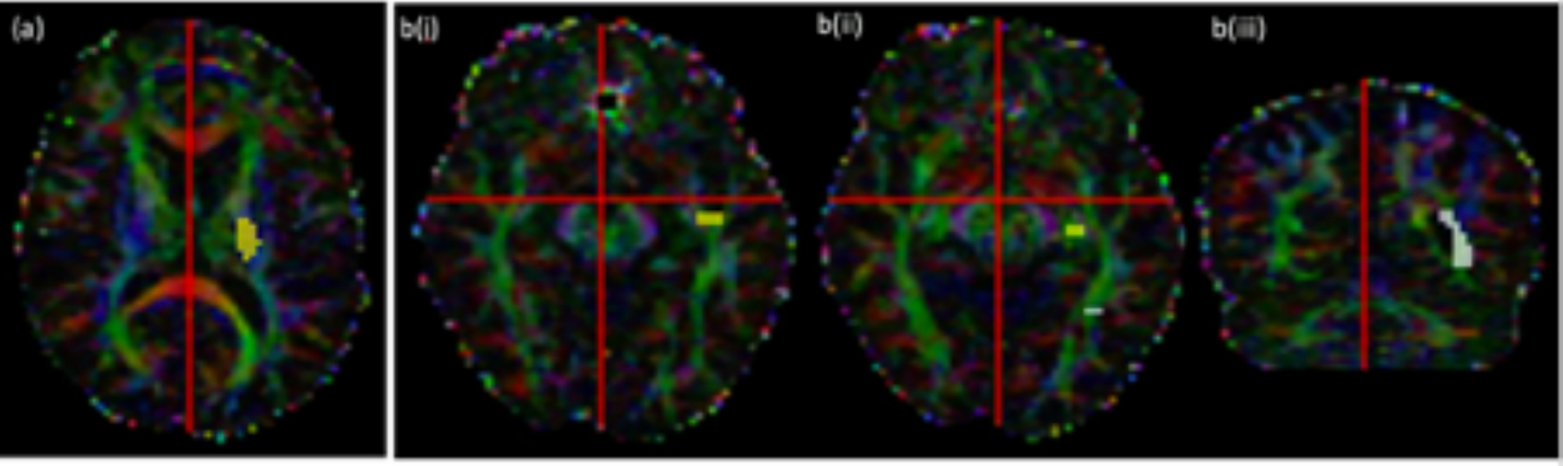
Volunteer subject tractography seed ROI’s (yellow) waypoint ROI’s (white) and exclusion masks (red) for (a) CST and (b) OR at 3 slice locations.

For the cortico-spinal tract a seed region was defined within the posterior limb of the internal capsule (PLIC), including only those voxels with diffusion predominantly in an inferior/superior direction as identified blue/purple on a colour coded fractional anisotropy map (*Fig 1*) [33].

For the optic radiation two seed regions were defined using the method described by [34]. The first contained voxels which were antero-lateral to the lateral geniculate nucleus (LGN) at the base of the Meyer’s loop with diffusion directions predominantly in an antero-medial to postero-lateral orientation (*Fig 1*B(ii)). The second was in the section of the Meyer’s loop (*Fig 1*B(i)). A waypoint was defined in the lateral wall of the occipital horn of the lateral ventricle at the posterior extent of the corpus callosum (*Fig 1* B(iii)). Using these ROI’s alone often results in the optic radiation including tracts from the inferior longitudinal fasciculus. To remove these erroneous pathways an objective, iterative process was performed on the tracts produced by CSD-based probabilistic tractography [34]. The process involved moving an exclusion mask posteriorly until it began to coincide with the Myers loop, leading to thinning of the optic radiation. The final location for the exclusion mask was chosen when 90% of the original tract volume remained, as determined through calculating the number of voxels in the resultant tract image. The tractography undertaken with this “90%” exclusion mask applied was assumed to be the best representation of the optic radiation. This exclusion mask was then applied to the tractography obtained from the other processing methods (as discussed below).

Seed and waypoint ROI’s definition files were copied from the STEALTH workstation ensuring that identical regions were used between the STEALTH and MRTrix-based processing. Therefore, any differences in tractograms could be attributed to the processing method rather than differences in ROI location.

The tractography process was terminated when streamlines entered voxels with a fractional anisotropy (FA) < 0.1 for DTI-based streamline tractography, with a fibre orientation distribution (FOD) amplitude < 0.1 for CSD-based streamline tractography, and an FOD amplitude < 0 for the CSD-based probabilistic tractography. The FA threshold in this study was set to 0.1 to ensure tractography was not prematurely terminated in regions of peritumoural oedema. For all tractography processing the maximum curvature was set to 45^o^ for the corticospinal tract (the default curvature setting for the StealthViz package) but was increased to 180^o^ for tractography of the optic radiation to ensure the high curvature tracts within the Meyer’s loop were not removed. Tractography step size for all algorithms was set at 1mm. In StealthViz the 3D object distance parameter was set to zero to ensure there was no artificial inflation of the tract volume.

For probabilistic tractography the output is a probability map with image intensities that corresponds to the number of streamlines passing through each voxel normalised to the total number of streamlines generated. These maps were thresholded such that voxels with probabilities less than 0.005, 0.01, 0.025, 0.05 and 0.075 had their value set to zero, and subsequently a binary map was created in NIfTI format for each threshold. Tractography outputs from the streamline methods were also converted to binary images, in which all voxels that were intersected by a streamline were considered to be part of that tract.

In total seven binary tract images were created corresponding to each processing method. These methods have been labelled as: (1) STEALTH (for DTI-based streamline tractography generated using the StealthViz package), (2) CSD-stream (for CSD reconstruction with streamline tractography generated using MRTrix), (3) Prob 0.005, (4) Prob 0.01, (5) Prob 0.025, (6) Prob 0.05 and (7) Prob 0.075 (for CSD reconstruction with probabilistic tractography generated using MRTrix, thresholded at 0.005, 0.01, 0.025, 0.05 and 0.075, respectively).

### Sub-study: CST tractography with waypoint

**Fig 2:**
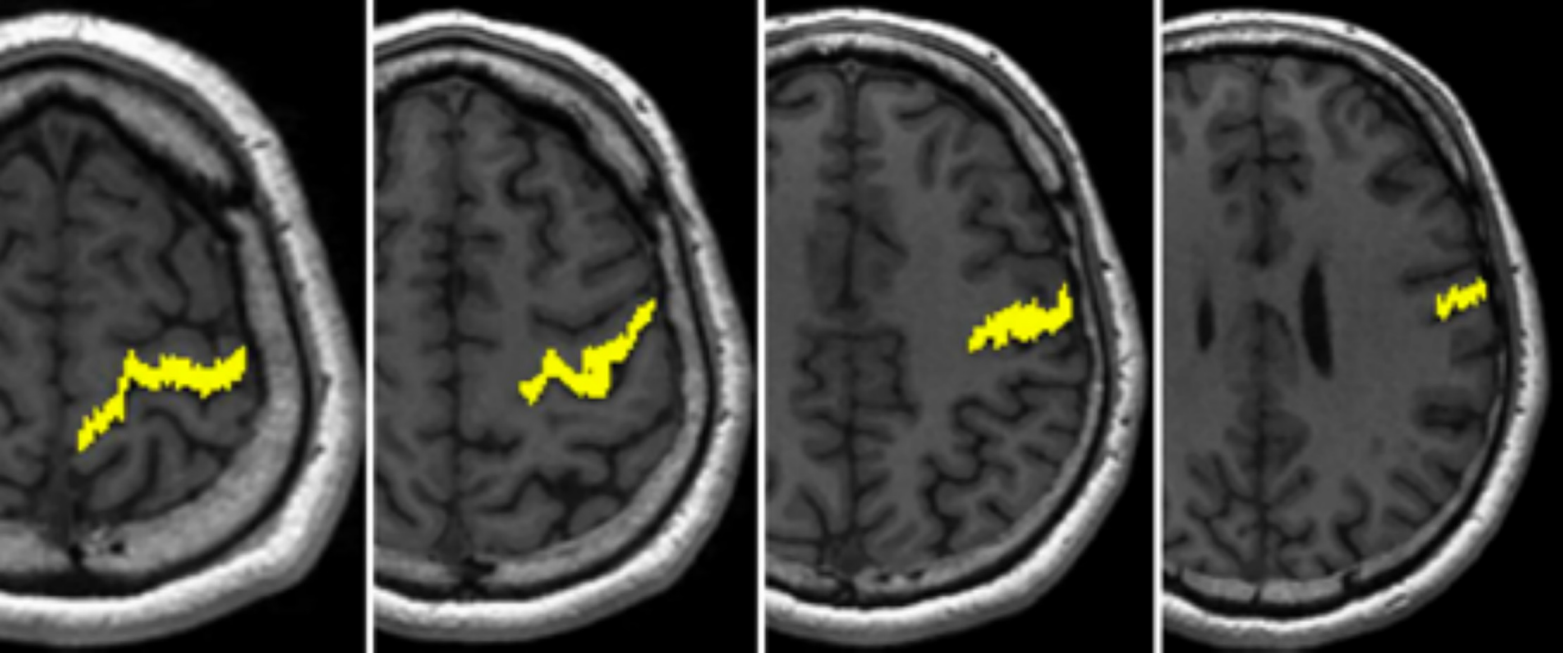
Precentral gyrus waypoint ROI for an example subject shown over a series of axial slices.

In addition, a “sub-study” was undertaken on a limited dataset with the aim of identifying if the inclusion of a waypoint ROI improved the tractography of the CST. The analysis was undertaken on the 32dir data (as it was expected that the outcome would not be overly dataset dependent) for the CSD-based probabilistic tractography processing using the PLIC seed ROI described above (*Fig 1*A) and a waypoint region placed in the precentral gyrus (*Fig 2*). Tracts were only included in the final probability map if they intersected this waypoint region.

### Tract comparison

#### Standard space

For the volunteer subjects, the diffusion images were aligned to their structural images using epi_reg (www.fmrib.ox.ac.uk/fsl/FLIRT) and the structural images were aligned to the Colin27T1 atlas using FNIRT (www.fmrib.ox.ac.uk/fsl/fnirt). The combined warp from epi_reg and FNIRT was then applied to the binary tract images, aligning these to the Colin27T1 atlas. The tracts were then summed for all 10 subjects to create a tract probability map. The tract probability map was compared to the Jülich atlas which is aligned to the same Colin27T1 atlas. The Jülich atlas consists of cytoarchitectonic maps of the white matter structures in the brain obtained from dissection studies [21]. The intensity of the voxels within these maps represents the probability that the voxel contains a particular tract based on 10 subjects. Comparisons with the Jülich atlas were performed both qualitatively, through visual inspection, and quantitatively by calculating the fuzzy Dice overlap scores [22]. The fuzzy Dice overlap quantifies the overlap of non-binary images whose voxel intensities vary across a fixed range, giving an overlap score in the range [0, 1]. In our case the two inputs were the combined subject tract probability map and the probability maps from the Jülich atlas.

#### Native space

The tract volume of the CST and OR for each subject was determined in their native space by summing the number of voxel in the binary tract image and multiplying by the voxel size. The Meyer’s loop to temporal pole (ML-TP) distance was measured for the OR only, and was determined from the difference in the anterior-posterior coordinate between the most anterior tip of the Meyer’s loop, (identified from tractography) and temporal pole as identified visually on the non-diffusion-weighted (b = 0) image. The accuracy of the ML-TP distance and the tract volume for the different methods was assessed by comparing to equivalent data obtained from published dissection studies [24, 25, 35, 36]

#### Statistical analysis

The data sets for tract volumes and ML-TP distance across the 10 volunteer subjects were tested for normality using the Shapiro-Wilks test. All data demonstrated a normal distribution and subsequently a one-way ANOVA investigation was undertaken to determine if the data could be considered to all be drawn from the same distribution. In cases where the data failed the ANOVA null hypothesis, a post-hoc Tukey honest significant difference (HSD) analysis was undertaken. Significance levels for all statistical testing was set to 0.05 and analysis was undertaken using MATLAB (Boston, USA)

### Clinical case studies

The investigation in our volunteer cohort suggested (see results section below) that the 64 direction acquisition and peripheral gating had limited impact on the quality of tractography. For our patient cohort we therefore implemented the 32 direction acquisition protocol. A qualitative comparison was undertaken comparing all seven processing methods. The fuzzy DICE overlap, ML-TP and tract volume measurements were not applied in the patient cohort since tissue distortion from the lesions would lead to expected misalignment with the atlas/dissection results.

## Results

### Tract comparison in standard space

Tracts were successfully depicted the CST in all subjects for all acquisition and post processing methods investigated. For the OR the CSD reconstruction with probabilistic tractography successfully depicted the pathway in all hemispheres. In 6 hemispheres CSD-based streamline tractography did not generate any tracts to depict the OR and in 4 hemispheres the DTI-based streamline tractography did not generate any tracts. This occurred for the 32 direction acquisition (1 hemisphere) and the 64 direction acquisition (3 hemispheres). These null results were included in fuzzy Dice overlap, and volume analysis but excluded from the analysis of the average ML-TP distance.

#### Dice overlap measure

**Fig 3:**
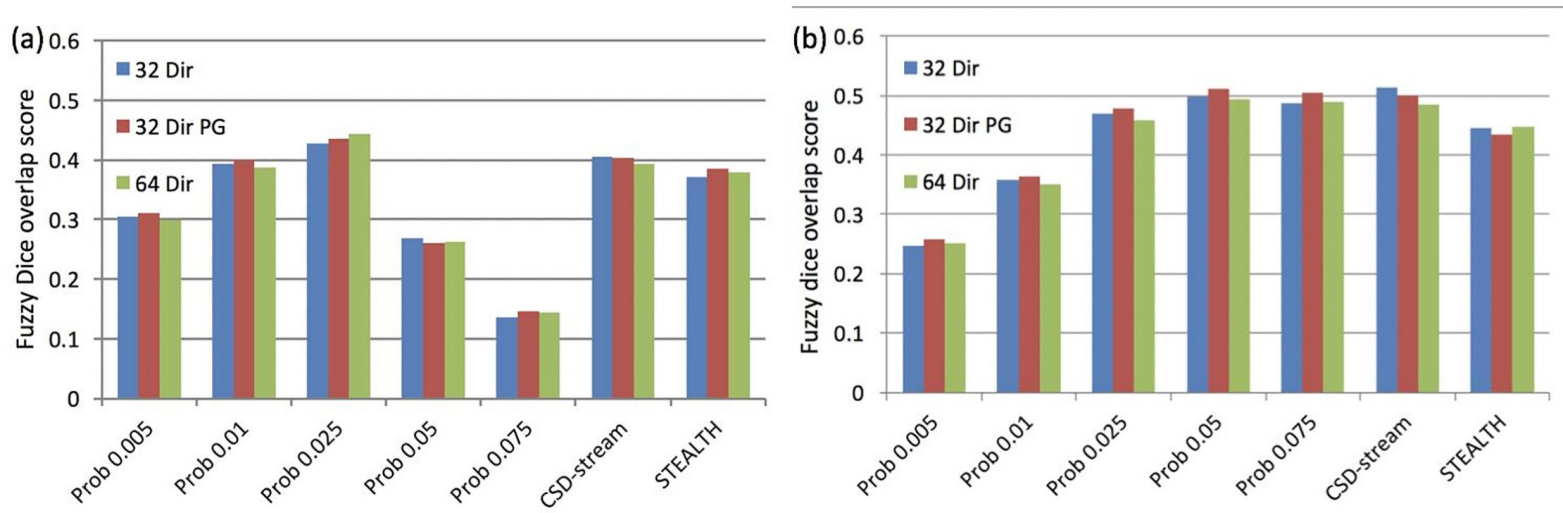
Fuzzy Dice overlap scores between the tractography and the Jülich atlas for (a) the CST and (b) the OR. Labels as per main text.

Results for the fuzzy Dice overlap analysis are shown in *Fig 3*, demonstrating only small differences in overlap between the tracts generated from the various acquisition protocols. Differences in overlap were more apparent between processing methods. It was found when averaging the overlap results from all 3 acquisitions that the CSD-based probabilistic tractography thresholded at 0.025 and 0.05 had the greatest overlap value (44% for the CST and 50% for the OR). When considering the three different processing methodologies (DTI-based streamline, CSD-based streamline and CSD-based Probabilistic at the optimal threshold) DTI-based streamline tractography generated by StealthViz had the poorest overlap with the Jülich atlas for both the CST and OR (38% and 44% respectively).

#### Qualitative comparison

**Fig 4:**
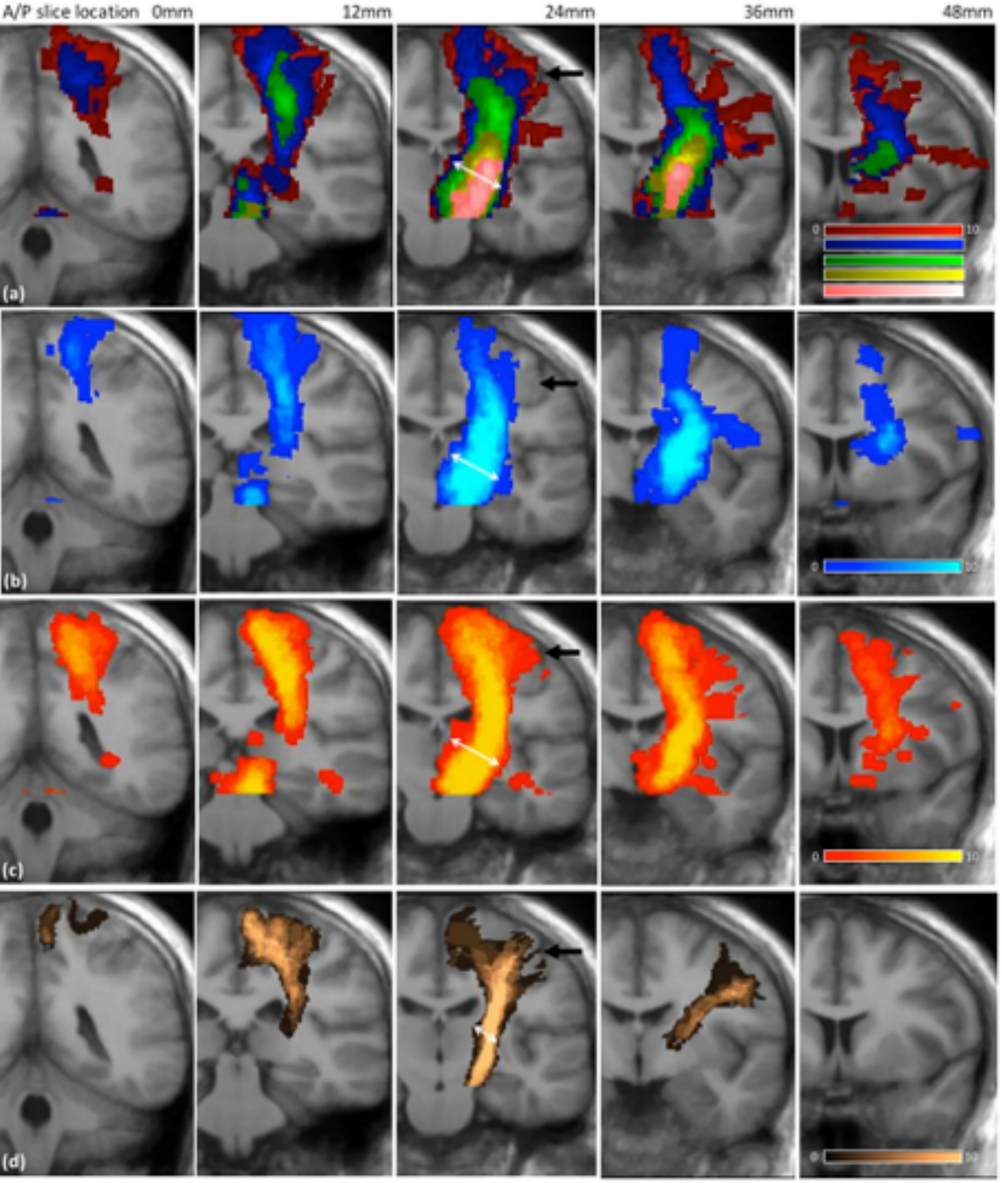
Tract frequency maps for the CST in standard space overlaid on the average structural image for all 10 volunteer subjects. Each column represents a different coronal slice location. Row (a) Prob 0.005 (red), Prob 0.001 (blue), Prob 0.025 (green), Prob 0.05 (yellow), Prob 0.075 (pink), Row (b) CSD-stream, Row (c) STEALTH, Row (d) Jülich atlas.

**Fig 5:**
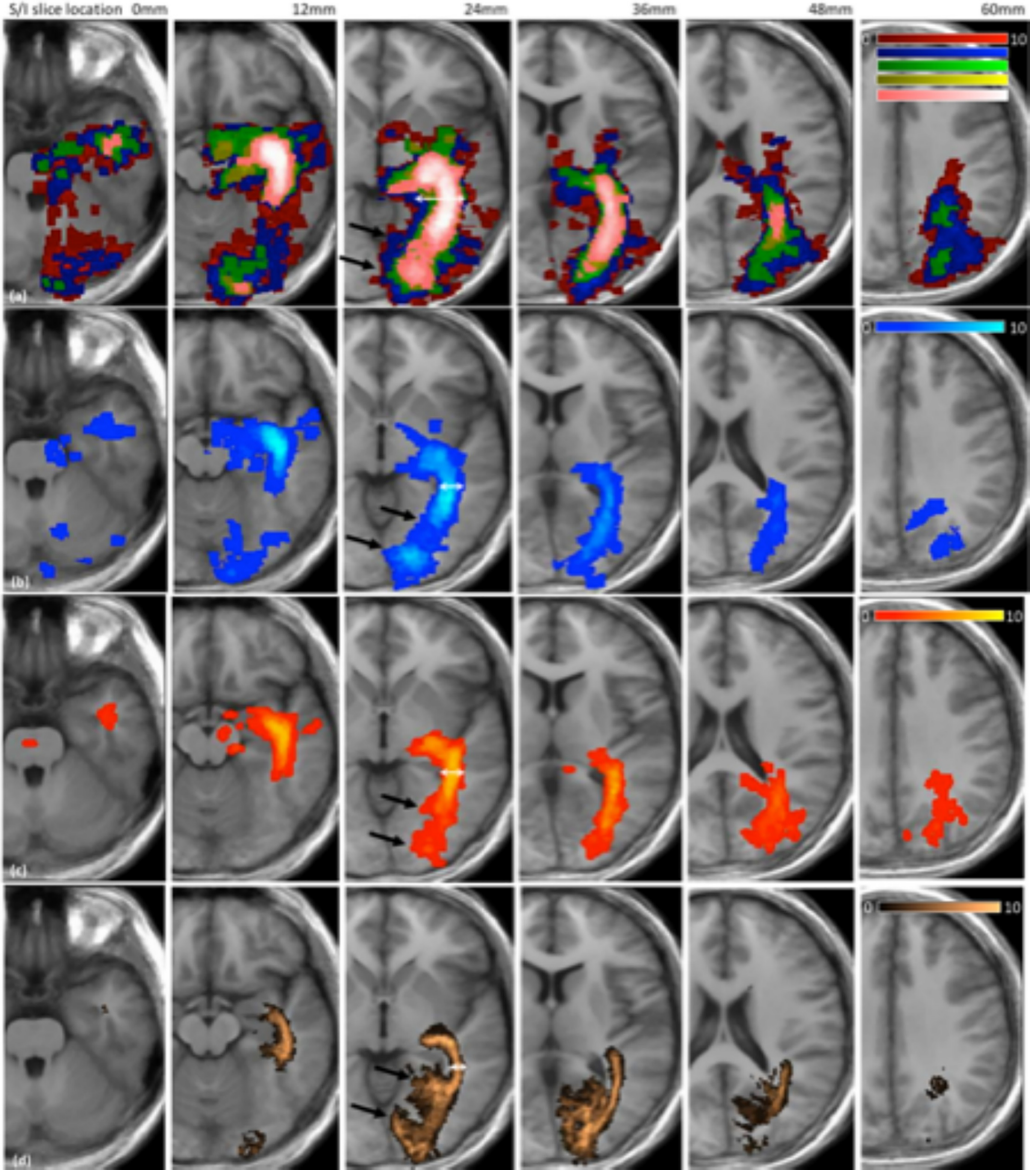
Tract frequency maps for the OR in standard space overlaid on the average structural image for all 10 volunteer subjects. Each column represents a different axial slice location. Row (a) Prob 0.005 (red), Prob 0.001 (blue), Prob 0.025 (green), Prob 0.05 (yellow), Prob 0.075 (pink), Row (b) CSD-stream, Row (c) STEALTH, Row (d) Jülich atlas.

Tract frequency maps for the CST and the OR are shown in *Fig 4* and *Fig 5* respectively, together with the Jülich atlas in standard space. The results are shown comparing processing methods only, since the overlap analysis highlighted that acquisition scheme had limited impact on tractography outcome (*Fig 3*). In all cases increasing the probabilistic threshold decreased the core thickness together with the total length of the depicted tract. However, the core tract thickness was typically overestimated by tractography in all methods relative to the size found in the Jülich atlas (see white arrows in *Fig 4* and *Fig 5*). Areas where tractography was limited in correctly depicting the underlying anatomy included the lateral projections of the CST (see black arrows *Fig 4*) and the anterior projections onto the calcarine fissure (indicated by black arrows on *Fig 5*).

### Tract comparison in native space

#### ML-TP distance

**Fig 6:**
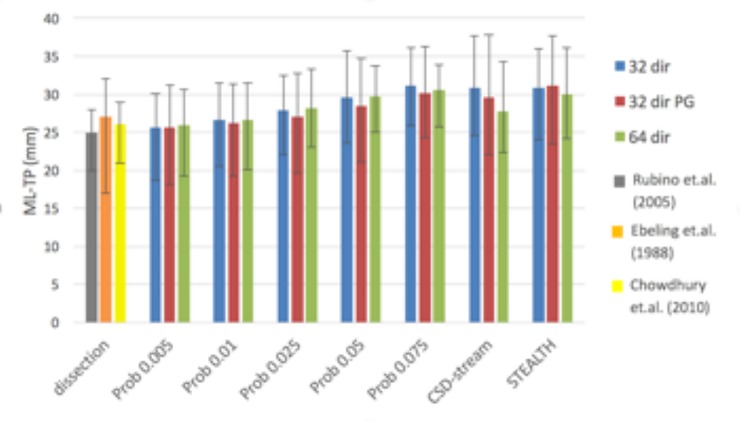
Average ML-TP distances for the optic radiation across 10 volunteer subjects compared against the three largest reported dissections studies. Error bars depict the ML-TP range.

Qualitatively, the ML-TP distance appeared to be most influenced by changing the probabilistic threshold, with the lower thresholds giving the best match to dissection studies (*Fig 6*). The data displayed limited variation between the acquisition methods and so statistical testing for significant differences in ML-TP distance was undertaken for the 3 processing methods: STEALTH, CSD-stream and Prob 0.05 for the 32 dir acquisition only. The Prob 0.05 was the only probabilistic threshold investigated in the statistical analysis since it produced the greatest overlap with the Jülich atlas when overlap values were averaged over the 3 acquisitions (see *Fig 3B*). The ANOVA investigation for these three groups highlighted no statistical significance between the ML-TP distance values.

#### Tract volume

**Fig 7:**
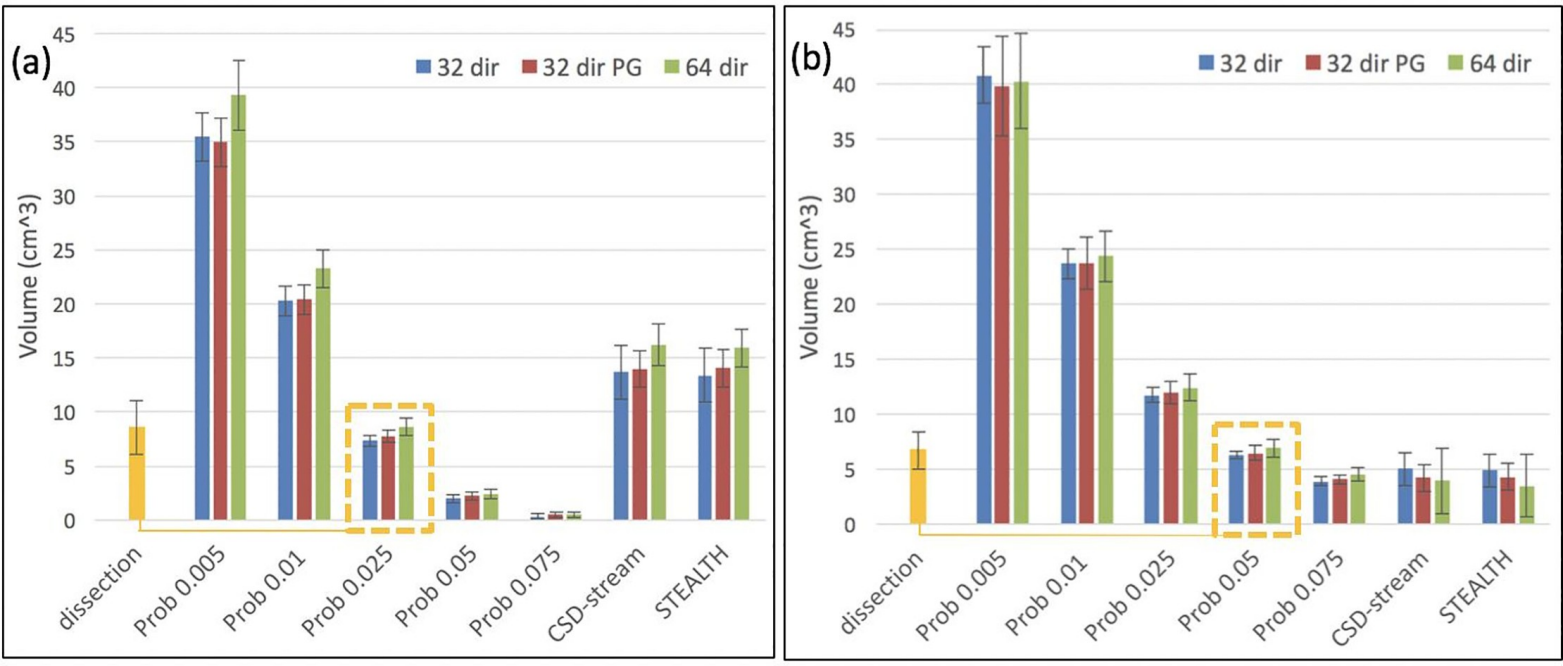
Tract volumes across 10 volunteer subjects for (a) the CST compared to dissection studies of [24] and (b) the OR compared to the dissection studies of [23]. Error bars depict the standard deviation in volume. The yellow dashed boxes highlight that Prob 0.025 and Prob 0.05 best match the results from dissections studies for the CST and OR, respectively.

Our tract volume measurements, shown in *Fig 7*, indicate that the Prob 0.025 and Prob 0.05 methods best matched the results of dissection studies for the CST and OR, respectively (*Fig 7* –yellow dashed boxes). The data displayed limited variation between the acquisition methods, therefore statistical testing to identify differences in tract volume was only undertaken between the 3 processing methods: STEALTH, CSD-stream and Prob (with a threshold of 0.025 for the CST and 0.05 for the OR) and for the 32 dir acquisition only. The 0.025 (CST) and 0.05 (OR) thresholds were chosen since these were identified as the optimal thresholds from the overlap analysis with the Jülich atlas when overlap values were averaged over all acquisitions. (see *Fig 3B*).

The ANOVA investigation for these three groups suggested that there were significant differences between the processing methods (p<0.01) for both the CST and OR tract volumes. Results of the post-hoc Tukey HSD are shown in *Table 2* where significant differences between methods are highlighted by an asterisk (p<0.01).

**Table 2:** Statistical comparison between methods for the 32 dir acquisition data only.

	CST	OR
STEALTH vs CSD-stream	p<0.01*	p = 0.064
STEALTH vs Prob†	p<0.01*	p<0.01*
CSD-stream vs Prob†	P<0.01*	p = 0.11

**†** The probabilistic tractography method was tested with a threshold of 0.025 and 0.05 for the CST and OR respectively.

* Comparisons which showed significance with threshold p<0.01

### Sub-study: CST tractography with waypoint

**Fig 8:**
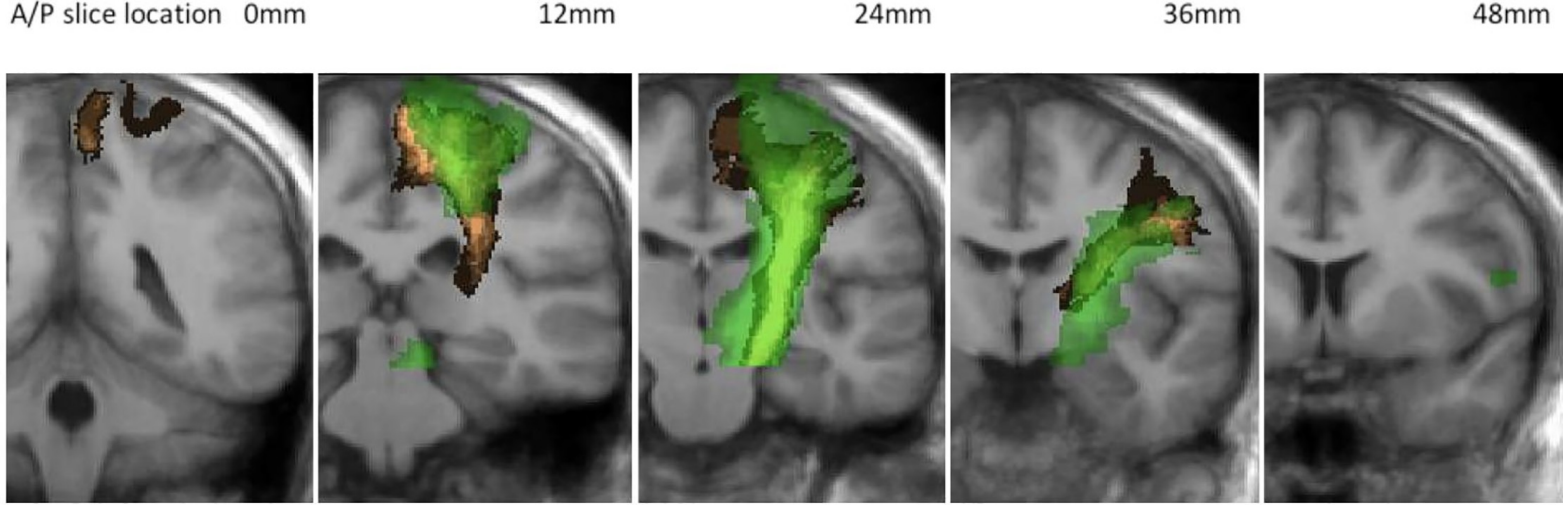
Tractography of the CST generated with the inclusion of a precentral gyrus waypoint ROI. Green: CST frequency map in standard space for Prob 0.025. Copper: CST defined in the Jülich atlas. Images are overlaid on the average structural image for all 10 volunteer subjects.

The sub-study investigated tractography of the CST for the optimal method from the volunteer results (32 direction acquisition without peripheral gating processed using the Prob 0.025), with the addition of a waypoint mask located in the precentral gyrus. The results shown in *Fig 8* highlight a fuzzy *Dice overlap score of 56%* and a volume of 10.81±0.46cm^3^ (mean±SD).

### Clinical case studies

#### Patient 1

**Fig 9:**
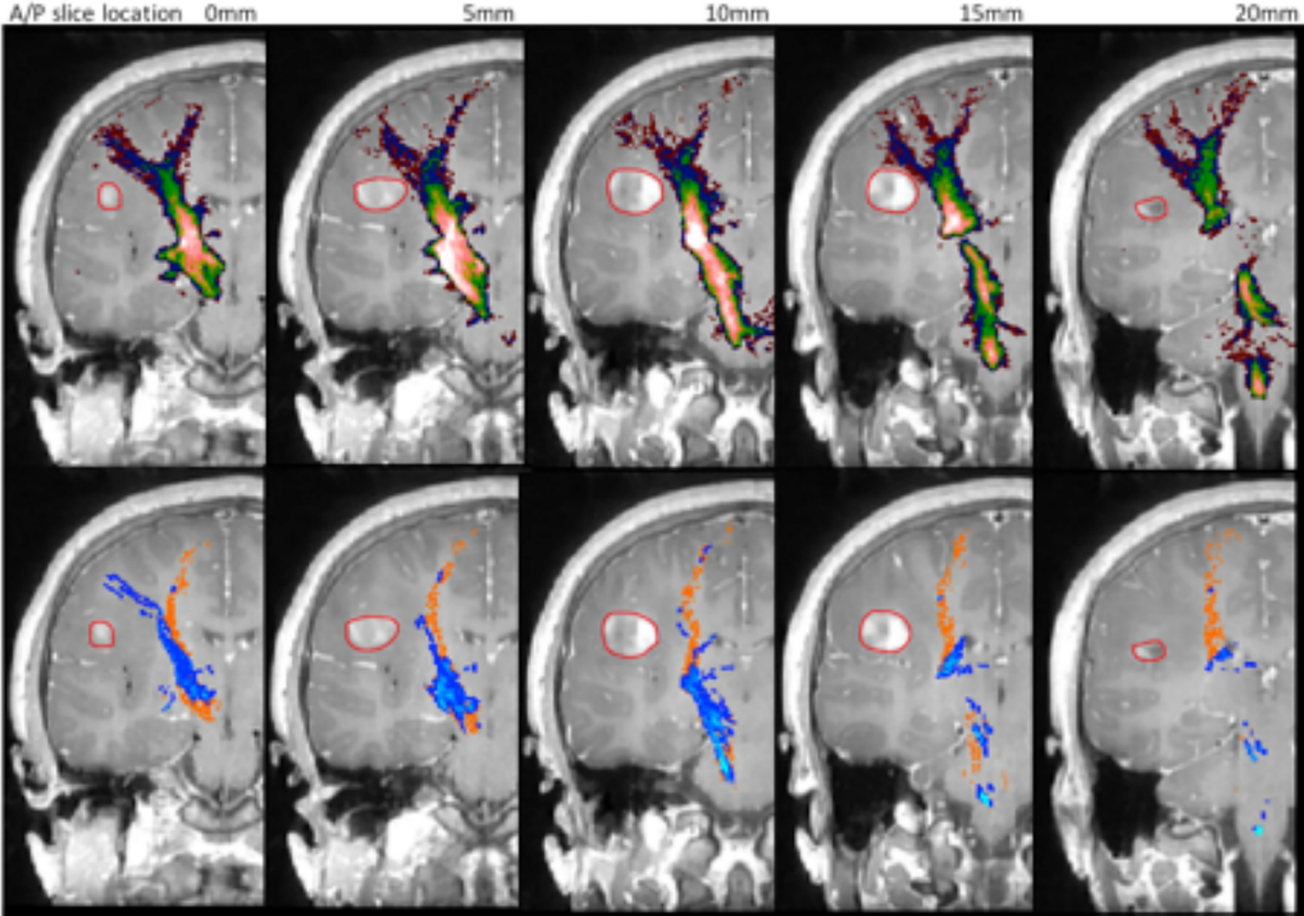
Tractography of the CST for patient 1. Top row: Prob 0.005 (red), Prob 0.01 (dark blue), Prob 0.025 (green), Prob 0.05 (yellow), Prob 0.075 (pink). Bottom row: CSD-stream (blue), STEALTH (orange). Lesion is outlined in red.

*Fig 9* indicates the results for patient 1. In the STEALTH tractography it can be seen that the lateral projections are not represented to the region of the precentral gyrus controlling hand function. They are; however represented for tractography based on the CSD reconstruction. For CSD-based probabilistic tractography at thresholds of 0.005 and 0.01 the lateral projections are well depicted; however the core of the CST is possibly over-represented as was the case in the volunteer cohort (*Fig 4*). At the optimal probabilistic threshold of 0.025 (as defined by the maximum tract overlap with the Jülich atlas in our volunteer cohort) the lateral projections are heavily “pruned” and at greater thresholds are no longer present.

#### Patient 2

**Fig 10:**
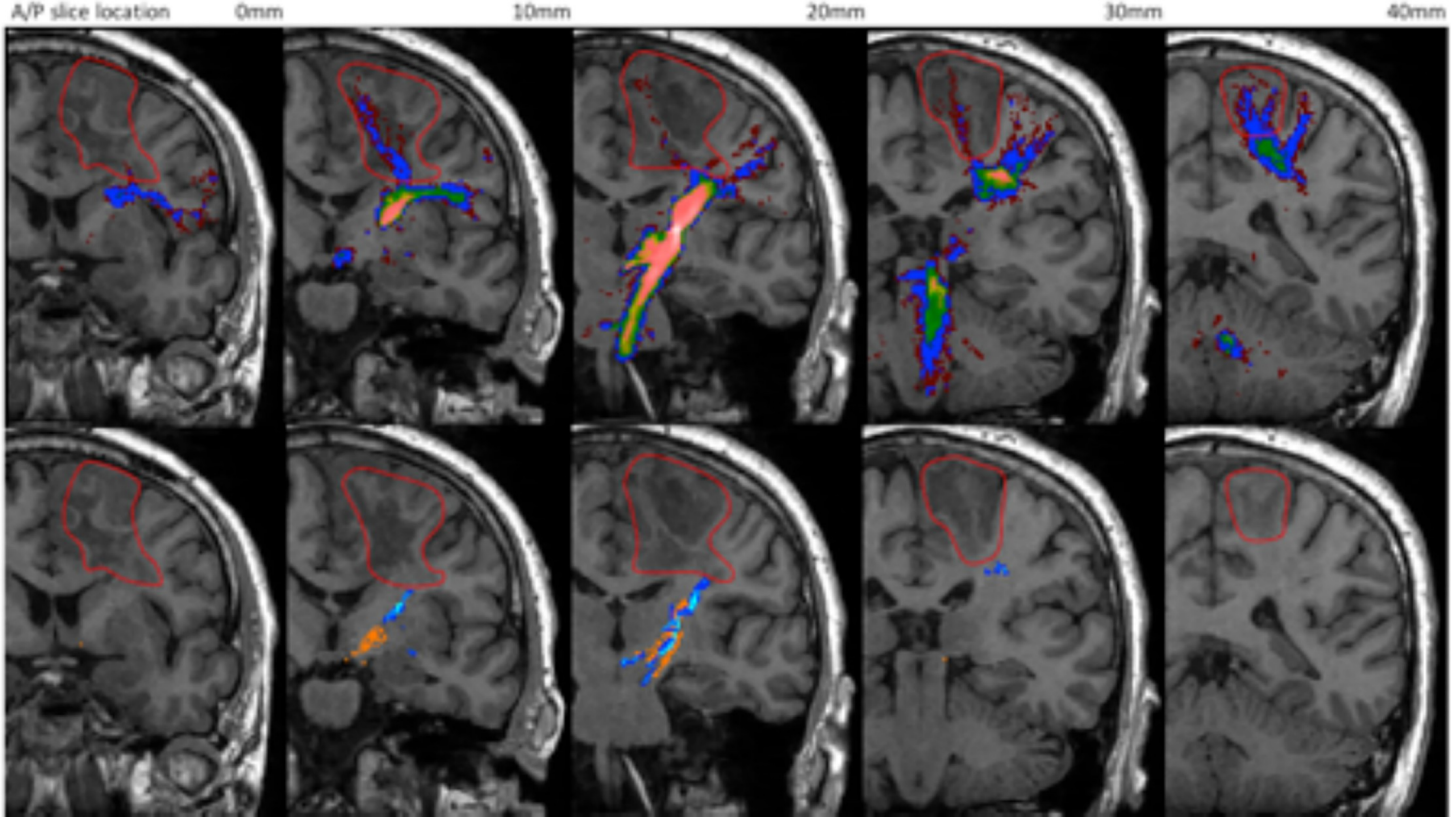
Tractography of the CST for patient 2. Top row: Prob 0.005 (red), Prob 0.01 (dark blue), Prob 0.025 (green), Prob 0.05 (yellow), Prob 0.075 (pink). Bottom row: CSD-stream (blue), STEALTH (orange). Lesion is outlined in red.

For Patient 2 the lesion included significant peri-tumoural oedema which potentially affected the CST. The CSD-based probabilistic method for the lowest threshold of 0.005 was able to delineate nearly the entirety of the CST connecting the PLIC to all regions of the precentral gyrus (*Fig 10*). As the threshold was increased the connectivity to the trunk area of the pre-central gyrus dominates, and projections to the foot and the face areas are progressively removed. Probabilistic tractography at all thresholds; however, provided better delineation of the CST compared to the streamline based methods (i.e CSD-stream and STEALTH).

#### Patient 3

**Fig 11:**
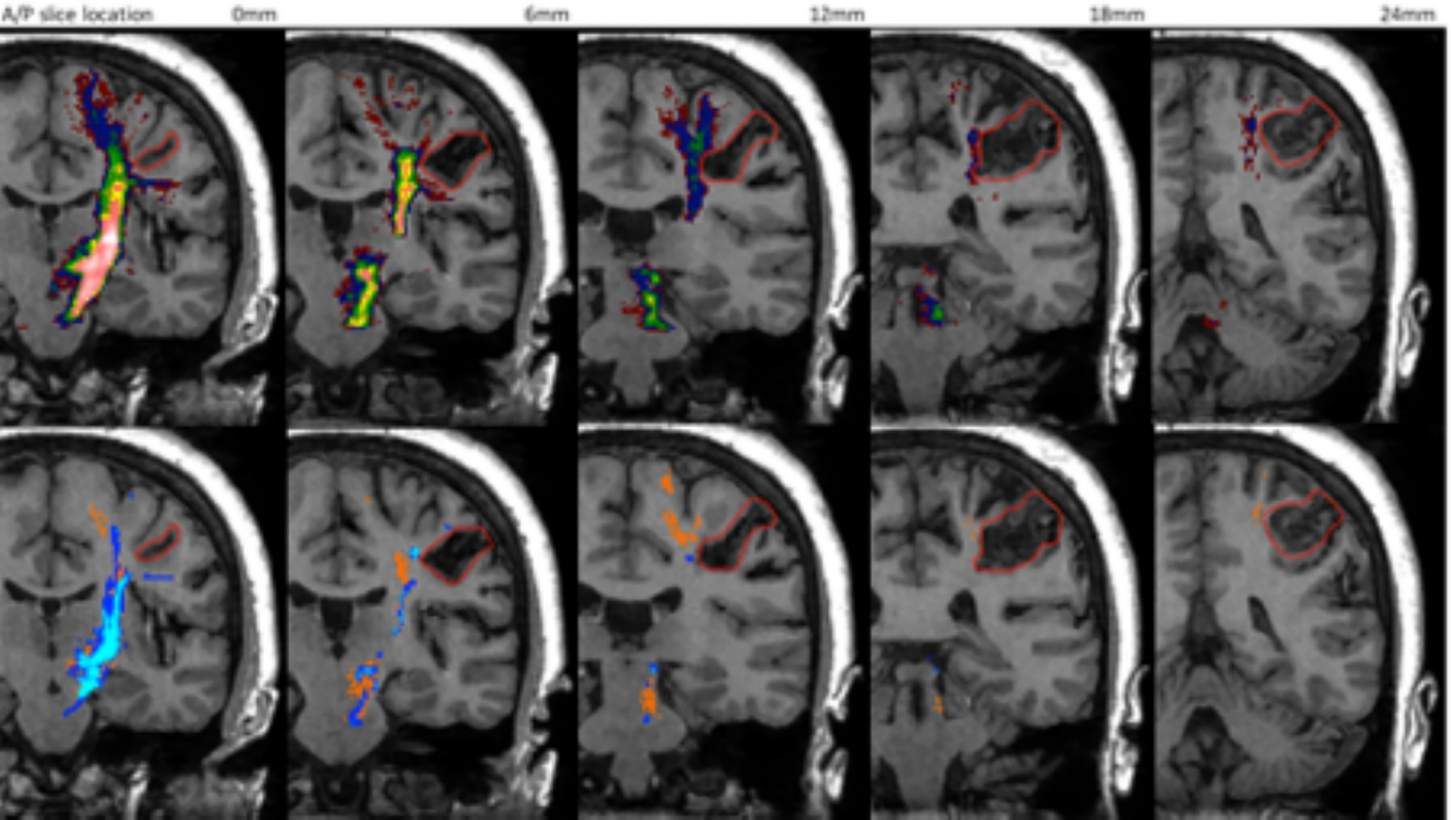
Tractography of the CST for patient 3. Top row: Prob 0.005 (red), Prob 0.01 (dark blue), Prob 0.025 (green), Prob 0.05 (yellow), Prob 0.075 (pink). Bottom row: CSD-stream (blue), STEALTH (orange). Lesion is outlined in red.

For Patient 3, the CSD-based probabilistic method with a threshold less than 0.025 produced tracts depicting a fuller extent of the CST (short arrow *Fig 11*). However, at these low thresholds it is likely that false positive tracts are present originating at the PLIC but ending near the superior parietal lobule (long arrow *Fig 11*). Both sections are immediately adjacent to the AVM but only the section depicted by the shorter arrow need be considered in the context of pre-surgical planning for treatment of the AVM.

## Discussion

The goal of this work was to optimise and assess the limitations of clinical tractography through a visual and quantitative comparison with dissection studies. The dissection metrics investigated included tract probability maps, tract volumes and the ML-TP distance for the optic radiation. By considering all known dissection metrics we aimed to gain the most comprehensive understanding of the accuracy of tractography in depicting the CST and OR relevant for our application of pre-surgical planning of space occupying lesions.

### Tract comparison in standard space

A qualitative assessment of tractography from our volunteer cohort displayed consistent false positive and false negative regions (*Fig 4* and *Fig 5*). The fuzzy Dice overlap measure, which quantifies these false negative/positive errors was found to be lowest for tracts produced using the StealthViz package (38% and 44% for the CST and OR respectively) and highest for tracts produced using CSD-based probabilistic tractography (44% and 52% for the CST and OR, respectively). The benefits of using the advanced processing methods is well documented and our results agree with published work which suggest moving beyond DTI methods for neurosurgical planning [13, 14, 17, 37]. The lower overlap value in the CST compared to the OR can partly be attributed to the use of a single seed ROI rather than implementing seed and waypoint ROI’s which help constrain tractography [38]. Many previous clinical studies investigating tractography of the CST for pre-surgical planning have similarly used only seed ROI’s [39–42]. This can be attributed to (a) difficulties in delineating an entire pathway between seed and end regions in the presence of peritumoural oedema [43, 44] and (b) difficulties in defining a waypoint ROI in patients where SOL’s can distort tissue [33, 45]. In our volunteer cohort; however, we undertook a sub-study to identify the effect of a waypoint ROI on tractography of the CST. We found that the fuzzy Dice overlap score increased from 44% (seed region only) to 56% (seed and waypoint) with a corresponding improvement in the qualitative visual similarity between the tractography and the Jülich atlas (*Fig 8*). This result highlights that the use of a waypoint ROI may be preferred in a clinical population if the operator can confidently identify the anatomy.

For tractography of the OR we chose multiple seed and waypoint ROI’s as per [34, 46] A large variety of seeding methods have been reported in the literature [47], perhaps the most common method being a single seed region directly adjacent to the LGN [48–50]. We investigated this seeding approach but found in several subjects the StealthViz algorithm produced tracts which did not follow the anatomical course of the optic radiations resulting in zero tracts with a waypoint region applied. Applying a whole brain tractography seed may have increased the likelihood of achieving connectivity between the seed and waypoint regions [3]. With whole brain tractography the pathways can be subsequently pruned to identify only those which maintain a connection between the seed and waypoint regions. However, in our study this approach would then have been inconsistent with the approach applied for the probabilistic based processing method.

For the OR, two previous studies have quantitatively compared tractography results to the Jülich atlas as a ‘gold standard’ [16, 51]. These studies used a modified receiver operating characteristics (ROC) analysis to obtain an objective threshold which optimises the overlap between tractography and the Jülich atlas for three consecutive coronal slices within the core of the OR. This threshold is then assumed to be optimal for the entire OR. Our fuzzy Dice overlap analysis is similar in concept, but optimises the threshold for the entire tract rather than a sub-set of slices. Unfortunately, a direct comparison of our optimal threshold with that reported by Clatworthy et al. (2010) was not possible since they defined their threhsold through the tract false positive rate (the optimal threshold being 2.5%) rather than a probability value as is typically done in probabilistic tractography.

### Tract comparison in native space

The ML-TP distance as reported in dissection studies had a value which best matched our results for the Prob 0.005 processing method (*Fig 6*). At the optimal probabilistic threshold of 0.05 (defined by the maximum tract overlap with the Jülich atlas) the ML-TP distance increased such that the tractography underestimated the anterior extent of the Meyer’s loop. A similar result was obtained from the CSD-stream and STEALTH processing methods. This inability to depict the full anterior extent of the Meyer’s loop is a known limitation of tractography of the optic radiation which has been previously reported in many studies [48, 49, 52]. This highlights the trade-off when choosing a probabilistic tractography threshold. To ensure the best representation of the Meyer’s loop the threshold should be lowered, with a corresponding potential increase in false positive pathways in other regions of optic radiations. The threshold should therefore be chosen to match the clinical application. If preservation of the Meyer’s loop is essential, then a low threshold is preferential. If optimal depiction of the complete optic radiation is preferred, as might be the case for neurosurgical planning for SOLs, then an intermediate threshold may be best.

The dependence of the ML-TP distance with tractography method and the qualitative relation of tractography of the optic radiation to the Jülich atlas was previously investigated by[17]. Similar to our results they reported no significant difference in ML-TP distance between diffusion tensor based tractography and probabilistic tractography with a threshold of 0.05. Measurements of ML-TP distance have also recently been investigated by [51], for CSD-based probabilistic tractography implemented using the MRTrix package (as we report here). In their study, they report a mean ML-TP distance of 30mm (range 20-34mm), which is greater than that reported in dissection studies. A direct comparison between our work and the work of Lim et. al. is difficult since they apply a threshold defined by a false positive rate of 2.5% (as discussed above). Their value of ML-TP distance (30mm) best matches ours for a probabilistic threshold of 0.075; however, it should be noted that seed and waypoint regions differ between our study and theirs.

Similar to our fuzzy Dice overlap analysis and ML-TP distance results, we found that the tract volume was minimally influenced by acquisition type whilst the effect of processing method and probabilistic threshold was significant (*Fig 7*). The tract volumes from the Prob 0.025 and Prob 0.05 provided the closest match to volumes reported in dissection studies for the CST and OR, respectively. It is interesting to note that these methods were also optimal for the fuzzy Dice overlap analysis with the Jülich atlas which suggests this may be the best approach for analysis of tractography in our clinical population. It should be noted; however, that these methods and thresholds may lead to an unacceptable false negative rate, particular in the presence of peritumoural oedema. In a clinical population this may correspond to eloquent pathways potentially not being represented in the output image.

The volume of the OR has been investigated in previous tractography studies [15, 53–55], which report a variety of values. The lowest reported value of 3cm^3^ was obtained using DTI-based streamline tractography [54, 55]. This closely matches the results we report from our STEALTH neurosurgical planning system which utilises a similar algorithm. [15] investigated the tract volume for single tensor probabilistic tractography. For a threshold of 0.05 they report an OR tract volume of approximately 5±1cm^3^ (mean±SD), whereas we report a slightly higher tract volume at the same threshold (6.6±0.6cm^3^ mean±SD). This difference can potentially be attributed to our use of a CSD-based reconstruction rather than a diffusion tensor reconstruction. Such increases in tract volumes have been qualitatively [13, 14, 29] and quantitatively [37] compared for DTI- and CSD-based tractography. Our data also confirms that tract volume is highly dependent on probabilistic tractography threshold, an important aspect which is far from standardized across the literature [34, 51, 53]. In the studies of [16] and [51], which utilised the modified ROC analysis to define an objective probabilistic threshold the volume was determined to be 16cm^3^ and 28cm^3^ respectively, values which are greater than the volumes we report and those published in previous dissection studies [23, 24]

### Clinical case studies

The 3 patients in this study were chosen to highlight the potential advantages and shortfalls of tractography from the various processing methods.

For patient 1, tractography based on CSD reconstruction was able to resolve the branching fibre structure of the CST to depict the lateral projections to the face/lips area of the precentral gyrus. Similar benefits of advanced tractography have been previously demonstrated in brain tumour patients [13, 14, 29, 37].

In patient 2, CSD based probabilistic tractography was the most successful at depicting the expected extent of the CST connecting the PLIC with the precentral gyrus. Both the CSD-stream and STEALTH methods produced pathways which terminated prematurely at the inferior aspect of the lesion. The success of probabilistic over streamline methods can potentially be attributed to difficulties in tracking through regions of peritumoral oedema surrounding the lesion in this patient [43, 44]. We implemented streamline tractography (STEALTH, CSD-stream) with a low FA/FOD threshold of 0.1 to facilitate tracking but still found the reconstruction of the pathway between the PLIC and precentral gyrus was disrupted. In the CSD based probabilistic method, an FOD threshold of zero was applied and the resulting tracts contained anatomically plausible connections but with a large number of false positive pathways. Through the application of a probabilistic threshold, we were able to prune many of the false positive tracts whilst still maintaining many of the expected connections between the PLIC and precentral gyrus.

The CSD probabilistic method thresholded at 0.025 (the optimal threshold from the overlap analysis with the Jülich atlas) potentially provided a good false positive/negative compromise for which the overall anatomical representation was better than that found with the streamline methods. A result which agreed with findings from a similar comparison [29].

In patient 3, pathways were depicted medial to the lesion for all processing methods; however, those generated using CSD-based probabilistic tractography at thresholds less than 0.025 produced likely false positive pathways which could result in an overly conservative surgical approach.

It should be noted that all three cases demonstrate that the optimal method from the volunteer study (Prob 0.025) still leads to an under representation of certain pathways (e.g. the lateral projections of the CST). This highlights how care should be taken when applying such thresholds in a clinical population where the presence of oedema can have a significant influence. A number of approaches have attempted to address this issue through modelling the free water component of the diffusion signal [56, 57] or through applying a generalised q-sampling acquisition scheme which allows for better modelling of both magnitude and direction of crossing fibres within a voxel [27]. These techniques identify regions of peritumoral oedema and improve the visualisation of tracts often though at the sacrifice of increased acquisition time.

### Limitations

A major limitation of this study is that the Jülich histological atlas defines tracts using a different methodology and on a different patient cohort. Patients who contributed to the Jülich atlas were considered to have normal brains (i.e. no chronic neurological or psychiatric disorders) but were of a different age range (37-85yrs) to our cohort. Although we do not expect this age difference to result in significantly different tract volumes and locations, any differences in these groups may limit the validity of our results. We undertook our studies on a 1.5T system using an 8 channel head coil. At other centres neurosurgical planning scans are often undertaken using a 3T system with head coils containing a larger array of elements resulting in improved image signal to noise ratio (SNR). Having results from such systems would be the focus of future work; however, we do not believe acquiring data on such systems would lead to significantly different results. Our comparison of 32 vs 64 directions should similarly produce an SNR increase and it was found, for these two acquisition schemes, there was limited differences in our results. In our study a single b-value was chosen which was not optimal for either DTI or CSD processing. An additional data set with optimal b-values for each method would be desirable but this was not possible within our allowed acquisition time. Instead, it was decided to focus on the number sensitising directions and peripheral gating as the acquisition variants. A single b-value of 1500 was chosen as a compromise to provide sufficient diffusion contrast for CSD processing whilst maintaining image SNR. A larger b-value would be preferred for the CSD technique improving the ability to resolve multiple fibre populations and a lower b value would be preferred for DTI improving the directional accuracy of the single fibre population. It has previously been shown; however, that both reconstruction methods can be undertaken at a b-value of 1500 [13]. Our volunteer cohort was limited to 20 hemispheres and a greater number would have been preferred in order to obtain a more robust conclusion and improved power for the statistical analysis. Finally, we investigated 3 patients using the optimised protocol from our volunteer studies. Investigating in further patients with more varied pathology is the subject for future studies. It should also be noted that in this study we focused on neurosurgical planning in patients with space occupying lesions only. Our results would most likely not translate to other neurosurgical applications such as stereotactic treatment for patients with tremor.

### Conclusion

The goal of this work was to understand where best to focus efforts in developing a robust clinical tractography program for pre-surgical planning of space occupying lesions. To this end we investigated clinically realistic adaptations to a standard diffusion acquisition protocol and compared an established clinical tractography tool (StealthViz) against a research tool (MRTrix), which is feasible to implement in a clinical setting; MRtrix processing time was approximately 3 minutes. It should be noted; however, that MRTrix, as well as many of the other software tools utilised in this study, are not CE marked for clinical use. In our volunteer cohort we found that the diffusion acquisition protocol had limited influence on tractography outcome compared to the processing method. When validated against the Jülich atlas and other dissection studies CSD-based probabilistic tractography was found to be the optimal method. Given this, for our clinical protocol we have implemented a 32 encoding direction diffusion acquisition scheme without peripheral gating, processed using CSD-based probabilistic tractography. In patients we found that this advanced methodology was able to delineate the branching fibre structure of the CST and that tracts were better depicted in regions of increased oedema where there is often reduced diffusion directionality. The results of our volunteer study; however, showed that despite the close correspondence between tractography and dissection-based volume measurements, the overlap measure to the Jülich atlas remained at best approximately 50%. This limitation highlights the trade-off between over-estimating the core of the tract volume and underestimating the peripheral tractography projections. This is particularly relevant when choosing a threshold for probabilistic tractography since this balance between false positive and false negative tracts is critically important for patient studies.
